# Suppressing Jahn–Teller Distortion in Manganese Oxides for High-Performance Aqueous Zinc-Ion Batteries

**DOI:** 10.3390/ma18122817

**Published:** 2025-06-16

**Authors:** Jiangfeng Duan, Man Huang, Ming Song, Weijia Zhou, Hua Tan

**Affiliations:** 1 Institute for Advanced Interdisciplinary Research (iAIR), School of Chemistry and Chemical Engineering, University of Jinan, Jinan 250022, China; duanjiangfeng1998@163.com (J.D.); ifc_zhouwj@ujn.edu.cn (W.Z.); 2 School of Materials and Chemical Engineering, Xuzhou University of Technology, Xuzhou 221018, China

**Keywords:** manganese oxides (MnO_x_), aqueous zinc-ion batteries (AZIBs), Jahn–Teller distortion (JTD), structural stability, electrochemical performance

## Abstract

Manganese oxides (MnO_x_) have been confirmed as the most promising candidates for aqueous zinc-ion batteries (AZIBs) due to their cost-effectiveness, high theoretical capacity, high voltage platforms, and environmental friendliness. However, in practical applications, AZIBs are hindered by the Jahn–Teller distortion (JTD) effect, primarily induced by Mn^3+^ (t_2g_^3^e_g_^1^) in octahedral coordination, which leads to severe structural deformation, rapid capacity fading, and poor cycling stability. This review systematically outlines the fundamental mechanisms of JTD in MnO_x_ cathodes, including electronic structure changes, lattice distortions, and their side effects on Zn^2+^ storage performance. Furthermore, we critically discuss advanced strategies to suppress JTD, such as cation/anion doping, interlayer engineering, surface/interface modification, and electrolyte optimization, aimed at enhancing both structural stability and electrochemical performance. Finally, we propose future research directions, such as in situ characterization, machine learning-guided material design, and multifunctional interfacial engineering, to guide the design of high-performance MnO_x_ hosts for next-generation AZIBs. This review may provide a promising guideline for overcoming JTD challenges and advancing MnO_x_-based energy storage systems.

## 1. Introduction

The growing demand for large-scale and sustainable energy storage systems has driven extensive research into safe, cost-effective, and environmentally friendly battery systems. Currently, lithium-ion batteries (LIBs) dominate the market due to their long cycle life, high energy density, and superior specific capacity, making them the preferred choice for large-scale energy storage and electric vehicles [1,2,3,4]. However, their widespread adoption faces significant challenges, including the scarcity of lithium resources and safety concerns associated with flammable organic electrolytes. Therefore, new energy storage systems are urgently needed to meet the demand for sustainable and eco-friendly energy systems.

Among these, aqueous zinc-ion batteries (AZIBs) have emerged as a promising candidate for LIBs due to their high theoretical capacity (820 mAh g^−1^ and 5855 mAh cm^−3^), high energy density, low cost, eco-friendliness, and ease of recycling [5,6,7,8]. Among various cathode materials, manganese oxides (MnO_x_) have emerged as one of the most promising candidates owing to their cost-effectiveness, high theoretical capacity, multiple oxidation states, environmental benignity, and abundant natural reserves [9,10,11,12]. However, in practical application, MnO_x_ cathodes in AZIBs are significantly hampered by the Jahn–Teller distortion (JTD) effect, which originates from the electronic configuration of Mn^3+^ (t_2g_^3^e_g_^1^) in octahedral coordination, resulting in elongation or compression of Mn-O bonds, and thus, inducing asymmetric lattice distortions and destabilizing the host structure [13,14,15,16]. Although Mn (IV) can undergo multi-electron transfer processes to deliver higher capacity, during the discharge process, partial reduction of Mn (IV) to Mn (III) occurs alongside disproportionation reactions (2Mn^3+^ → Mn^2+^ + Mn^4+^) and exacerbate the capacity fading. When MnO_2_ serves as the cathode material, the dissolution of Mn^2+^ induces structural collapse in the crystal framework, resulting in diminished ion storage sites. Concurrently, Mn^2+^ dissolution reduces active material content, thereby compromising the cathode’s reversible capacity. Therefore, the JTD effect will trigger a severe structural collapse, compromised cyclability, and deteriorate Zn^2+^ diffusion kinetics [17,18,19]. Nowadays, to address the challenges, extensive efforts have been devoted to suppressing JTD through strategies such as heteroatom doping (e.g., cation, anion, or oxygen vacancies), interlayer engineering, surface modification, and electrolyte optimization [20,21,22,23,24,25]. These approaches aim to stabilize the MnO_x_ framework, enhance intrinsic conductivity and charge/mass transport efficiency, and improve Zn^2+^ storage reversibility.

In this review, we systematically elucidated the mechanism of Jahn–Teller distortion in MnO_2_, influencing factors, and demonstrated adverse effects on the structure while analyzing how these structural changes affect electrochemical performance. Then, we introduced current common strategies for suppressing Jahn–Teller distortion and finally outlined future directions for mitigating this phenomenon.

## 2. Fundamentals of Jahn–Teller Effect in MnO_x_

Jahn–Teller distortion is an important phenomenon in transition metal oxides, particularly in MnO_2_-based materials, where structural deformation arises from the inherent electronic instability of specific transition metal ions and their tendency to minimize energy in octahedral coordination environments [26,27,28,29,30]. As a cornerstone of crystal field theory, this effect explains how Mn^3+^-containing octahedral complexes (t_2g_^3^e_g_^1^ configuration) undergo spontaneous symmetry breaking through tetragonal distortion to achieve system stabilization. The distortion originates from the degeneracy of e_g_ orbitals (dz^2^ and dx^2^ − y^2^) in high-spin Mn^3+^ (d^4^) ions, where asymmetric electron occupation triggers lattice deformation to lift orbital degeneracy and lowers the overall energy state [1,31,32,33,34].

In MnO_2_ cathodes for aqueous zinc-ion batteries, this effect manifests through three critical aspects. Firstly, the electronic structure, the labile e_g_ electron in Mn^3+^, induces unequal Mn-O bond lengths [35], such as elongation and/or compression. Secondly, structural consequences trigger irreversible phase transitions (layered → spinel) and MnO_6_ octahedra tilting [36]. Ultimately, electrochemical impact accelerates Mn dissolution via disproportionation and deteriorates Zn^2+^ diffusion [37]. The aforementioned aspects will be discussed in detail below.

### 2.1. Electronic and Structural Basis

**Figure 1:**
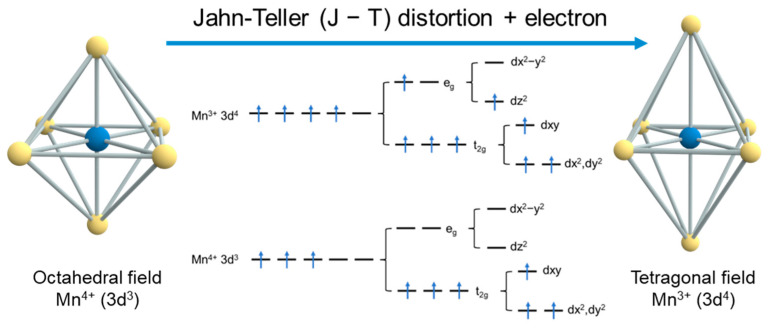
Mn 3d orbital energy level splitting in an octahedral field [43].

In manganese dioxide (MnO_2_), the Jahn–Teller (JT) effect plays a pivotal role in determining the electronic and structural properties, which arises from the electronic instability of Mn^3+^ (3d^4^) ions in octahedral coordination, and thus significantly influencing their electrochemical behaviors [38]. Universally, the valence state of Mn is +4 in MnO_2_. However, during battery operation, the cathode gains electrons, and the valence of Mn is reduced from Mn^4+^ to Mn^3+^. For Mn^3+^ (3d^4^), under the influence of an octahedral crystal field with sixfold coordination, the 3d orbitals split into two energy levels [39,40]: t_2g_ (triply degenerate, lower energy) and e_g_ (doubly degenerate, higher energy). According to the Aufbau principle, the four 3d electrons preferentially occupy the lower-energy t_2g_ orbitals before filling the higher-energy e_g_ orbitals [41,42]. As illustrated in Figure 1 [43], three electrons occupy the t_2g_ orbitals, while the remaining electron resides in the e_g_ orbitals. The e_g_ orbitals (comprising dz^2^ and dx^2^ − y^2^) host only one electron, creating an asymmetric electron distribution [44,45]. This imbalance induces directional shielding effects: the dz^2^ orbital provides stronger shielding along the axial (z-axis) direction. The dx^2^ − y^2^ orbital exhibits reduced electron shielding in the equatorial plane compared to the dz^2^ orbital, which would create the asymmetry of the electron and establishes unequal electrostatic repulsion between Mn^3+^ ions and the surrounding oxygen atoms [46,47]. Therefore, this imbalance drives the characteristic Jahn–Teller distortion, where the MnO_6_ octahedron undergoes either axial elongation or compression to achieve a lower-energy configuration. During the electrochemical reduction of MnO_2_, the conversion from Mn^4+^ to Mn^3+^ promotes this distortion process, then elongates the Mn-O bonds along the y-axis direction [48,49]. The resulting structural deformation generates substantial localized lattice strain that accumulates with cycling, eventually leading to the degradation as follows: a microstructural collapse that exposes fresh active surfaces to the electrolyte, initiation of the Mn^3+^ disproportionation reaction (2Mn^3+^ → Mn^2+^ + Mn^4+^ [50]) at the electrode–electrolyte interface, and irreversible phase transformation to the thermodynamically stable spinel-type MnO_2_. Finally, the capacity will fade due to both active material loss and increased charge transfer resistance, originating from the dissolved Mn^2+^ species migrating into the electrolyte solution and structural reorganization of the remaining Mn^4+^ [51].

This comprehensive degradation mechanism highlights the critical interplay between electronic structure (orbital asymmetry), atomic-scale structural changes (Jahn–Teller distortion), and macroscopic electrochemical performance in MnO_x_ cathodes. The understanding of these coupled phenomena provides essential insights for developing stabilization strategies targeting each degradation pathway.

### 2.2. Impact of Jahn–Teller Effect on Electrochemical Implications in AZIBs

Based on this fundamental understanding, we will investigate the correlation between Jahn–Teller distortion effects and the electrochemical performance of MnO_2_ cathodes in AZIBs [13,14,52]. As one of the most promising host materials for AZIBs, MnO_2_’s intrinsic properties govern the performance by the following key aspects such as the specific capacity, determined by accessible redox-active sites and their utilization efficiency, cycling stability, dictated by structural integrity, and the rate capability, influenced by ion transport kinetics [17,53,54,55].

The charge-storage mechanism in MnO_2_ mainly relies on the reversible insertion/extraction of Zn^2+^/H^+^ ions [56]. However, during the reversible process, strong electrostatic interactions with the host lattice will be created during migration due to the substantial ionic radius and high charge density of Zn^2+^. These interactions aggravated the inherent Jahn–Teller distortion of Mn^3+^-containing octahedra, with each electrochemical cycle progressively accumulating lattice strain. This cyclic distortion ultimately leads to manganese dissolution via disproportionation and structural collapse through bond-length fatigue, both contributing to capacity fading [57,58,59,60]. The specific capacity, cycling performance, and dissolved Mn^2+^ comparison of the modified MnO_2_ cathode materials of aqueous Zn-ions batteries are summarized in Table 1.

Jahn–Teller effect impacts capacity through the following interrelated mechanisms [68]. First, manganese dissolution reduces the quantity of electroactive material [69]. Then, lattice distortion decreases the accessibility of remaining Zn^2+^ storage sites by modifying coordination environments [70,71]. Finally, the induced strain field disrupts ion migration pathways, increasing the activation energy for Zn^2+^ diffusion in severely distorted regions, which can be confirmed by recent density functional theory calculations [72].

In conclusion, the structural evolution studies reveal that the Jahn–Teller effect drives irreversible phase transitions in MnO_2_ polymorphs, which reduce both Zn^2+^ storage sites and diffusion coefficients by an order of magnitude, consistent with the phenomenon observed experimentally. The cumulative Jahn–Teller distortion during cycling initiates a cascade of degradation processes in MnO_2_ cathodes, including local lattice strain accumulation, manganese dissolution, structural collapse, and detrimental phase transitions. These interrelated phenomena collectively limit the cycle life and restrict the practical capacities of MnO_2_ in most reported systems. Currently, the mitigation strategies mainly focus on orbital engineering through transition metal doping and strain–relief structural designs, which will be discussed in subsequent sections.

## 3. Mitigation Strategies to Suppress the Jahn–Teller Effect

To overcome these limitations, strategic modification of MnO_2_ has become imperative, with the central objective of stabilizing the crystal lattice against Jahn–Teller-induced deformations.

**Figure 2:**
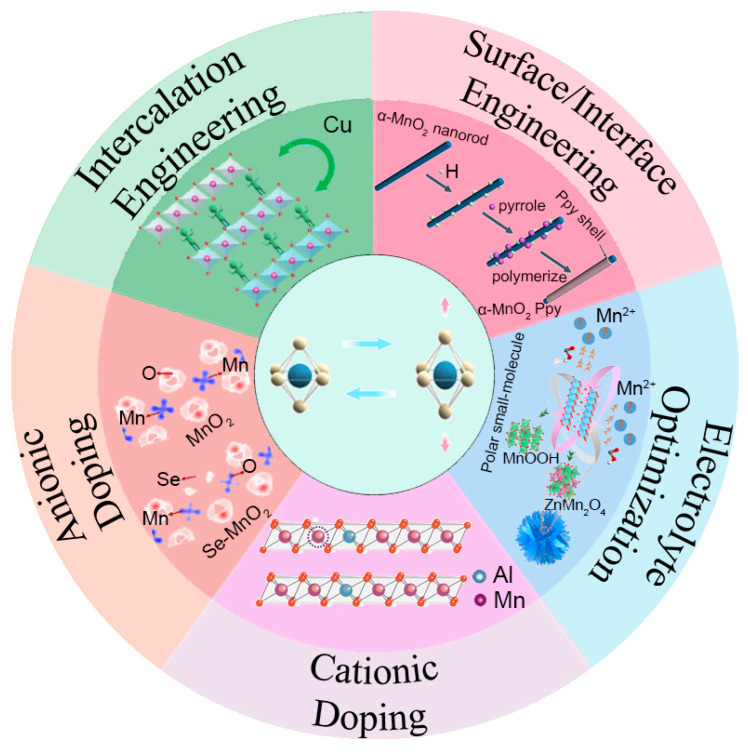
Schematic illustration of strategies to suppress the Jahn–Teller effect [61,63,76,77,78]. Ref. [77] Copyright 2020, Elsevier. Ref. [78] Copyright 2025, Elsevier.

Figure 2 shows the currently reported modification strategies to suppress the Jahn–Teller effect mentioned. It focuses on the following fundamental aspects of the distortion mechanisms: strengthening the main structure through cationic/anionic doping [73], electronic structure modulation to reduce e_g_ orbital degeneracy, and interface stabilization via surface engineering [74]. These strategies simultaneously address the root causes of Jahn–Teller effects and improve zinc-ion diffusion kinetics and charge transfer efficiency. Notably, recent advances also combine the above approaches with electrolyte optimization to promote synergistic stabilization effects [75]. This multi-dimensional modification paradigm will demonstrate promising improvement strategies toward advanced MnO_2_-based materials.

### 3.1. Strategic Cationic Doping for Structural Stabilization

**Figure 3:**
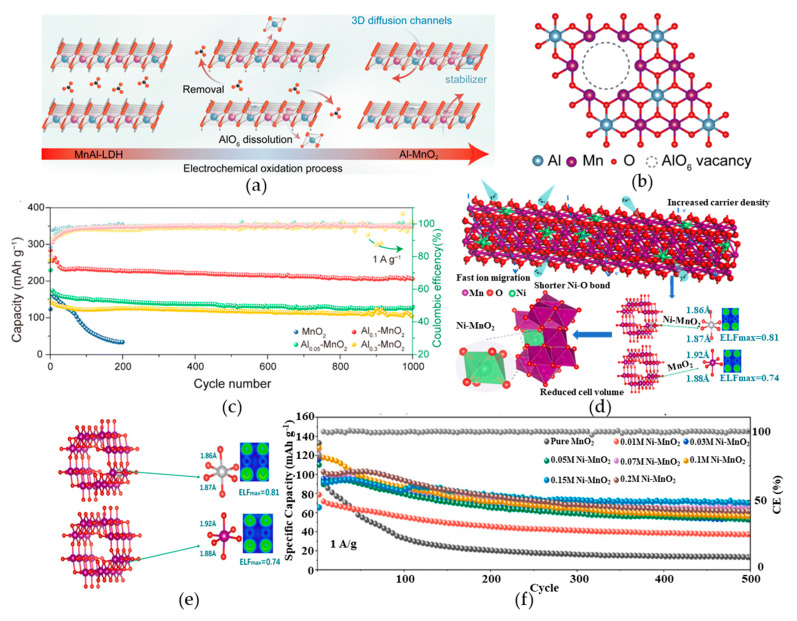
(**a**) Schematic illustration of the synthesis process for Al-doped MnO*_2_*. (**b**) Atomic structure model of Al_0.1_MnO_2_. (**c**) Comparative cycling performance of Al_0.1_MnO*_2_* versus pristine MnO*_2_*. (**d**) Schematic illustration of the structure model for Ni-doped MnO*_2_*. (**e**) Optimized structure and charge density localization diagram. (**f**) Cycle performance of MnO_2_ and Ni-MnO_2_. Ref. [61] Copyright 2024, Royal Society of Chemistry. Ref. [79] Copyright 2025, Elsevier.

Nowadays, to suppress the Jahn–Teller distortions and enhance the structural stability of the MnO_2_ based materials, cation doping has been confirmed as one of the most effective strategies, which fundamentally operates through the strategic substitution of Mn^4+^ in MnO_6_ octahedra with foreign cations, thus simultaneously modulates bond strength through altered orbital hybridization, optimizes the local crystal field environment, and regulates valence electron distribution to effectively suppress Jahn–Teller distortions. For example, Zhao et al. [61] employed electrochemical oxidation to synthesize Al-doped δ-MnO_2_ (Al_x_MnO_2_, x ≈ 0.1), where controlled Al^3+^ substitution created beneficial cation vacancies (Figure 3b) that enabled three-dimensional Zn^2+^ diffusion with reduced energy barriers while providing additional redox-active sites. In addition, the stabilization mechanism is confirmed through the comprehensive characterizations. First, inductively coupled plasma (ICP) analysis demonstrated a significant reduction in Mn dissolution. Second, density functional theory (DFT) calculations revealed a dramatic increase in Mn dissolution energy, indicating enhanced lattice cohesion. Third, the variation in Mn-O bond length during Zn^2+^ insertion was markedly suppressed, with the decrease in bond length, ultimately delivering exceptional electrochemical performance (327.9 mAh g^−1^ at 1 A g^−1^ with 92.4% capacity retention over 1000 cycles) (Figure 3c). In addition to Al^3+^ doping discussed in the aforementioned work, other cation dopants such as Ni also exhibit inhibitory effects on Jahn–Teller distortion. Liang et al. [79] prepared Ni-doped α-MnO_2_ (Ni-MnO_2_) as shown in Figure 3d. Compared to Mn^4+^, Ni^2+^ has a smaller atomic radius; its occupation in the lattice causes the surrounding O atoms to be arranged more closely, thereby Ni^2+^ doping resulting in shorter and more uniform chemical bond lengths compared to Mn-O bonds at equivalent positions. The electron localization function (ELF) diagram shown in Figure 3e also shows that the electron localization around the Ni^2+^ is enhanced compared to the Mn ion at the same position, indicating that the Ni-O has a stronger interaction and the material’s stability is improved. The analysis of the results is shown in Figure 3f. Ni-MnO_2_ has extremely excellent cycle stability. This optimized bond length effectively suppressed Jahn–Teller distortion, significantly enhancing the material’s cycling performance. Collectively, these findings validate the effectiveness of cationic doping in mitigating Jahn–Teller distortions while simultaneously enhancing both the capacity and cycling stability in AZIBs.

### 3.2. Anionic Doping for Enhanced Structural Resilience

**Figure 4:**
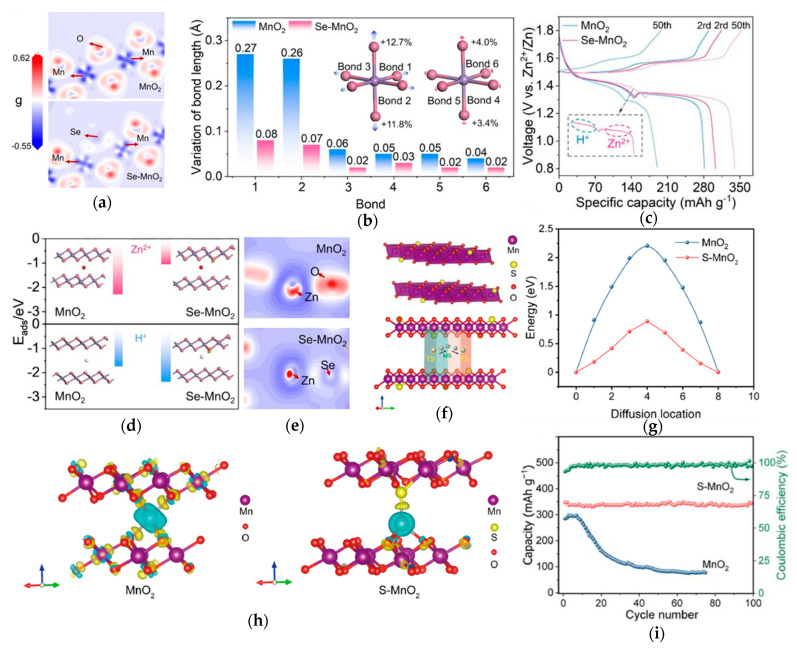
(**a**) Differential charge density analysis of MnO_2_ and Se-MnO_2_. (**b**) Changes in Mn-O bond length at the discharged state of MnO_2_ and Se-MnO_2_. (**c**) Cyclic performance (3.0 A g^−1^). (**d**) Adsorption energies of Zn^2+^ and H^+^ migration in MnO_2_ and Se-MnO_2_. (**e**) Differential charge density analysis of MnO_2_ and Se-MnO_2_ after Zn^2+^/H^+^ co-intercalation. (**f**) The atomic structure model of S-MnO_2_ and the side view of a schematic illustration of Zn migration in S-MnO_2_. (**g**) The Zn ion diffusion barrier profiles for MnO_2_ and S-MnO_2_. (**h**) Charge density difference distribution diagrams for Zn ion in MnO_2_ and S-MnO_2_. (**i**) Cycling performance of MnO_2_ and S-MnO_2_ electrodes at a current density of 200 mA g^−1^. Ref. [63] Copyright 2024, Wiley-VC. Ref. [80] Copyright 2025, Elsevier.

The incorporation of anionic species at the O sites of MnO_6_, attributed to the varying covalency of Mn-X interactions, where X represents an anion such as F^−^, S^2−^, and PO_4_^3−^, has also been confirmed as one of the most promising approaches to suppress the JTD and improve the electrochemical properties. By introducing these species, the distortion of MnO_6_ octahedra can be effectively suppressed. Thus, Ye et al. [63] explored the anion species of Se to mitigate Jahn–Teller distortions of MnO_2_ (Figure 4a). The mechanism of doping effect and inhibition of Jahn–Teller distortions are evidenced by comprehensive experimental and computational analyses. As shown in Figure 4e, the charge density differentials reveal a substantial weakening of Zn^2+^ host electrostatic coupling in Se-MnO_2_, directly correlating with reduced lattice strain and suppressed octahedral distortion. The stabilization effects are quantitatively demonstrated through comparative bond length analysis (Figure 4b), confirming effective suppression of both bond elongation and manganese dissolution, which are the hallmark manifestations of Jahn–Teller distortion. The stabilization mechanism of the doping effect to suppress the Jahn–Teller is primarily reflected through the following aspects: optimized ion adsorption energetics (Figure 4d) and inhibited H^+^ co-intercalation that typically exacerbates structural degradation. These synergistic improvements enhanced the electrochemical performance of Se-MnO_2_, which maintains 98.5% capacity retention over 1000 cycles and delivers 325 mAh g^−1^ reversible capacity, confirming that anionic doping is a potent strategy for developing stable MnO_2_ cathodes. Similar to the Se element doping, other anion dopants also substitute oxygen positions in the original [MnO_6_] octahedra. Zhao et al. [80] synthesized S-MnO_2_ (Figure 4f). The roles of these anion dopants in cathode materials are analogous: they regulate the electronic structure of MnO_2_ and weaken the electrostatic interactions between Zn^2+^ and the host material. As shown in Figure 4g, S-MnO_2_ has a lower diffusion energy barrier of Zn^2+^, and the charge density difference distribution in Figure 4h indicates the same result. In sharp contrast, the insertion of Zn in S-MnO_2_ possessed a smaller charge depletion area, indicating less electronic interaction between the inserted Zn^2+^ and S-MnO_2_. The capacity fading caused by Jahn–Teller distortion is mitigated (Figure 4i). The introduction of anion dopants primarily modulates the electronic structure while structurally exerting dual effects: weakening Zn^2+^-host electrostatic interactions and strengthening Mn-O bonds. These synergistic actions collectively suppress Jahn–Teller distortion and enhance cycling stability.

### 3.3. Interlayer Engineering for Structural Stabilization

**Figure 5:**
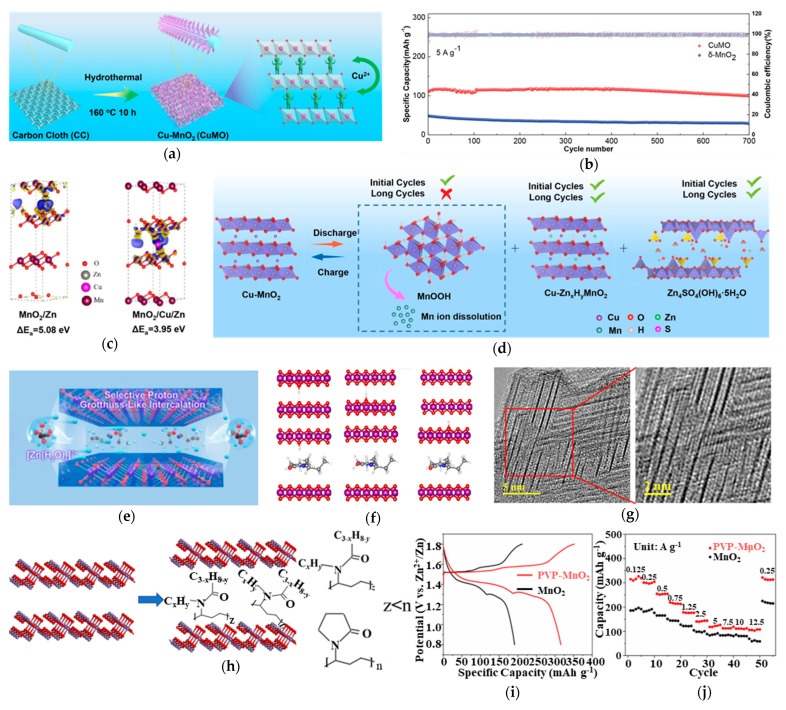
(**a**) Schematic illustration of the synthetic process of CuMO electrode. (**b**) Long-cycling performance (5 A g^−1^). (**c**) Formation energies of δ-MnO_2_ and CuMO. (**d**) Schematic illustration of the electrochemical reaction mechanism of Zn//CuMO batteries. (**e**) Schematic illustration of H^+^ and Zn^2+^ transport in PVP-MnO_2_ electrode. (**f**) Calculation model of H transmission state. (**g**) TEM images of PVP-MnO_2_. (**h**) Schematic diagram of PVP intercalation in δ-MnO_2_. (**i**) Galvanostatic charge/discharge profiles. (**j**) Rate performance of MnO_2_ and PVP-MnO_2_. Ref. [76] Copyright 2021, Elsevier. Ref. [84] Copyright 2023, Wiley-VC.

The strong electrostatic interactions between Zn^2+^ ions and the host lattice interlayers serve as a primary driver of Jahn–Teller distortion. Therefore, it is crucial to make efforts to mitigate these interactions to regulate the structural stability of the MnO_2_ host. Interlayer engineering has emerged as a multifaceted strategy to counteract Jahn–Teller distortions in MnO_2_ cathodes through simultaneous geometric confinement and electronic structure modulation. Among the various modulation strategies, the pre-intercalation of pillaring structures between the layers is an effective approach to simultaneously stabilize the interlayer framework and expand the interlayer spacing. Zhang et al. [76] demonstrated this concept by introducing Cu^2+^ pillars (CuMO) into the MnO_2_ structure, as shown in Figure 5a, achieving an expanded interlayer spacing of 0.7 nm. This structural modification yielded remarkable electrochemical performance, with CuMO delivering enhanced specific capacity and maintaining stable cycling over 700 cycles at 5 A g^−1^ in Figure 5b. The reduced Zn^2+^ insertion energy in CuMO (Figure 5c) directly confirms the weakened electrostatic interactions between the host structure and Zn^2+^ ions, facilitating easier ion intercalation. Additionally, the charge-discharge mechanism of CuMO (Figure 5d) reveals the mechanism of the Cu^2+^ pillars maintaining structural integrity during the electrochemical processes according to the stable lattice spacing observed before and after cycling. This dual functionality of Cu^2+^ pillars—both expanding the interlayer space and providing robust structural support—ensures the preservation of crystal structure integrity while simultaneously mitigating the detrimental effects of Jahn–Teller distortion. In addition to ion pillars, other species can also be confirmed as the ideal interlayer structures, such as Na^+^ [81], K^+^ [82], and PVA [83], et al. have also been demonstrated as effective interlayer engineering strategies for MnO_2_.

Beyond inorganic atoms, organic molecules also serve as effective interlayer pillars. PVP-pillared δ-MnO_2_ synthesized by Zhang et al. [84] in Figure 5e,g indicates PVP expanded the interlayer spacing and weakened the electrostatic interactions between Zn^2+^ and the host structure. Figure 5g shows a conspicuous terrace-shaped superlattice structure. In MnO*_2_*/MXene, the superlattice significantly reduced ion diffusion barriers, and the diagram of the PVP-MnO_2_ hybrid superlattice in Figure 5h shows the increased electron entropy triggers more t_2g_–e_g_ transitions for better reactivity, while the selective proton Grotthuss intercalation behavior is thus stemmed from the hybrid superlattice structure and optimized charge distribution. This inhibition of Zn^2+^ insertion provided high specific capacity (Figure 5i) and improved the rate ability of the material (Figure 5j).

### 3.4. Hierarchical Surface–Interface Stabilization Strategies

In practical applications, the MnO_2_ cathodes demand an integrated stabilization approach coordinating atomic-scale electronic modulation with macroscopic structural engineering to address the multiscale challenge of Jahn–Teller distortion in AZIBs. At the atomic level, surface doping induces orbital hybridization, which will reduce the e_g_ orbital splitting energy and prevent localized strain accumulation during cycling. Therefore, surface coating is one of the effective measures to simultaneously enhance the intrinsic conductivity and suppress the volume expansion/Mn dissolution to mitigate structural collapse caused by Jahn–Teller effects. Carbon-based materials have emerged as the most promising candidates for this application due to their exceptional electrical conductivity, remarkable structural versatility, and excellent cost-performance ratio.

**Figure 6:**
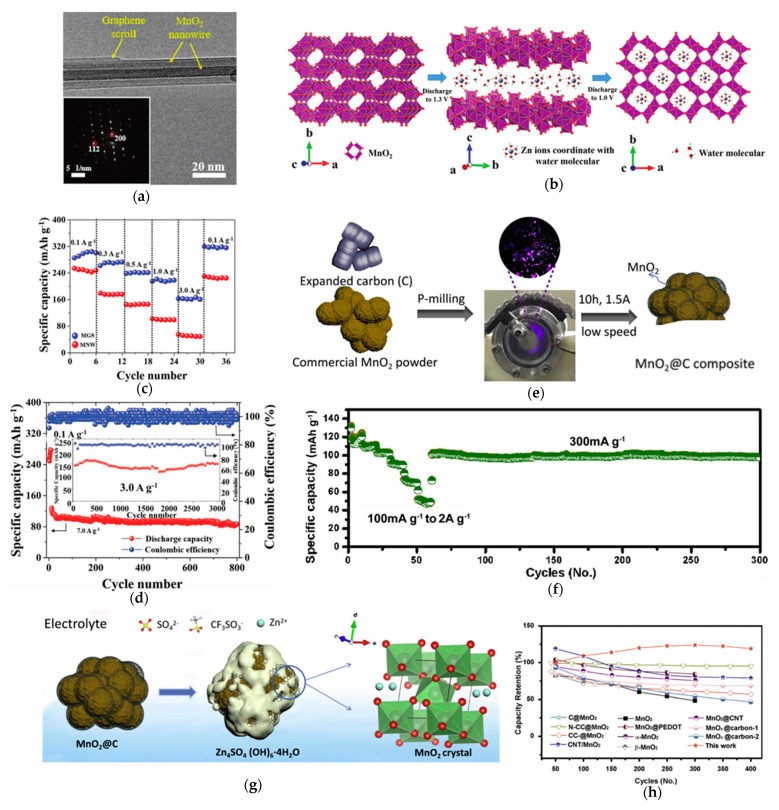
(**a**) TEM images (SAED inset in (**a**)). (**b**) Schematic illustration of the two-step intercalation mechanism of MGS cathode. (**c**) Charge and discharge curves of MGS at current densities ranging from 0.1 to 3 A g^−1^. (**d**) Long-term cycling performances at 7 and 3 A g^−1^ (inset). (**e**) Preparation processes of β-MnO_2_@C composites. (**f**) Rate performance at current rates from 100 mA g^−1^ to 2 A g^−1^. (**g**) Schematic showing the reactions during the discharge process for β-MnO_2_/Zn cell. (**h**) Cycle performance of β-MnO_2_@C. Ref. [62] Copyright 2018, Wiley-VCH. Ref. [85] Copyright 2018, Wiley-VCH.

Wu et al. [62] developed a graphene-coated MnO_2_ nanowire composite (MGS) through a controlled hydrothermal synthesis process (Figure 6a). The conformal graphene coating serves as both an efficient electron conductor and structural stabilizer. This core-shell architecture demonstrates excellent rate capability (89% capacity retention at 3 A g^−1^, Figure 6c), as well as outstanding cycling stability (92% capacity retention over 3000 cycles at 3 A g^−1^, Figure 6d).

The kinetic process was analyzed with galvanostatic intermittent titration technique (GITT), revealing a two-stage Zn^2+^ insertion mechanism and Figure 6b plays the two-step intercalation mechanism of MGS cathode: an initial rapid intercalation phase (DP I) with high diffusion coefficient at the MnO_2_/graphene interface, and a slower diffusion-limited phase (DP II) with Zn^2+^ penetrating into the 2 × 2 tunnels of α-MnO_2_. The kinetic transition at 1.30 V causes reactant accumulation, with DP II producing substantially greater lattice strain that typically triggers structural damage and Mn dissolution in bare MnO_2_. However, the graphene coating in MGS effectively suppresses these degradation pathways, as evidenced by a 78% reduction in Mn^2+^ dissolution compared to uncoated MnO_2_ nanowires.

Jiang et al. [85] prepared a β-MnO_2_ material coated with thin graphite films via a P-milling strategy, as shown in Figure 6e. The pores within β-MnO_2_ facilitated electrolyte infiltration, while the graphite-integrated structure significantly enhanced the material’s conductivity and suppressed Mn dissolution. Consequently, the β-MnO_2_@C cathode exhibited exceptional rate capability and cycling stability (Figure 6f). As illustrated in the discharge mechanism schematic (Figure 6g), during discharge, zinc ions precipitate within the pores between the graphite layers and β-MnO_2_. The graphite coating preserves the structural integrity of β-MnO_2_, maintains its ion storage sites, and ensures high capacity retention in β-MnO_2_@C (Figure 6h).

It suggested that the comprehensive protective mechanism, combining strain redistribution, electronic connectivity maintenance, and dissolution suppression, shows a generalizable modification approach, which can be successfully extended to other coating materials, including conductive polymers (e.g., PEDOT) and metal oxides (e.g., Al_2_O_3_). Therefore, the surface engineering strategies demonstrate the validity of stabilizing Jahn–Teller-active electrode materials in rechargeable battery systems.

### 3.5. Electrolyte Optimization in Suppressing Jahn–Teller Effect

In addition to the electron and structural factors, electrolyte engineering has also emerged as one of the effective strategies for mitigating Jahn–Teller distortions in MnO_2_ cathodes through interfacial chemistry control and bulk electrolyte modulation. Recent advances demonstrate that it can effectively stabilize the Mn^3+^ state through tailoring electrolyte formulations by modifying the solvation structure of Zn^2+^ ions and creating protective interfacial layers.

**Figure 7:**
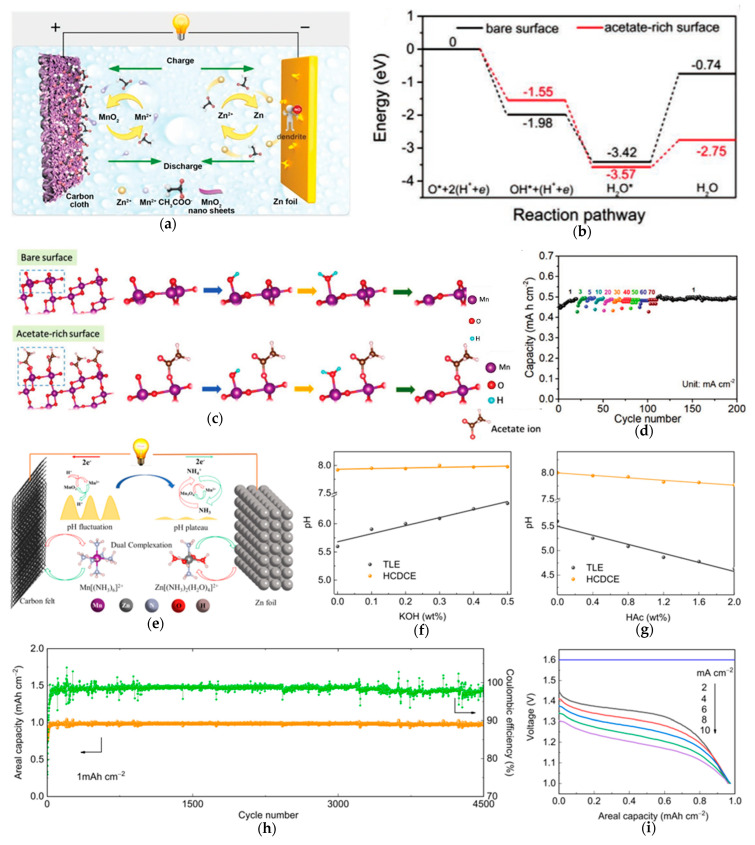
(**a**) Zn/MnO_2_ system design with acetate electrolyte. (**b**) Energy diagram of the dissolution reaction on MnO_2_ with a bare surface and with an acetate-rich surface. (**c**) Atomic structures for the dissolution reaction on MnO_2_ with a bare surface and with an acetate-rich surface. (**d**) Rate performance (1–70 mA cm^−2^) with acetate electrolyte. (**e**) Schematic illustration of the pH stabilization mechanism and reaction process of Zn–Mn electrolytic battery with the HCDCE electrolyte. The pH value changes with various additions of (**f**) KOH and (**g**) HAc into the electrolytes. (**h**) The long-term cycling performance at 2 mA cm^−2^ with a constant areal charge capacity of 1 mAh cm^−2^. (**i**) The rate performance at various current densities (2–10 mA cm^−2^). Ref. [86] Copyright 2020, Wiley-VCH. Ref. [87] Copyright 2021, Elsevier.

Acetate-based electrolytes, for instance, exhibit unique advantages through the formation of adsorption-induced passivation layer on MnO_2_ surfaces. For example, Zeng et al. [86] study the MnO_2_ charge/discharge mechanism (Figure 7a). The reaction pathway shifts from single-electron to two-electron transfer by replacing conventional sulfate anions with acetate groups, enabling direct Mn^4+^ → Mn^2+^ conversion and, thus, suppressing Jahn–Teller-active Mn^3+^ intermediates (Figure 7c). This change is mainly attributed to the modification effect of the acetates, as shown below. First, the H_2_O* adsorption energy is weakened (Figure 7b) while the Mn^2+^ discharge product is stabilized. Then, the transformed reaction kinetics yield exceptional electrochemical performance, including remarkable rate capability (Figure 7d) (70 mA cm^−2^ with minimal capacity loss) and ultra-stable cycling over 4000 cycles. The acetate electrolyte simultaneously addresses the challenges of eliminating Jahn–Teller distortion, suppressing manganese dissolution, and enhancing proton transfer kinetics, providing a new paradigm in aqueous zinc battery design.

Beyond suppressing Mn dissolution by adding Mn^2+^-containing salts, high-concentration electrolytes also effectively mitigate Mn dissolution. Huang et al. [87] developed a 46.5 M NH − Ac − NH_3_ − Zn(Ac)_2_ − Mn(Ac)_2_ buffered solution (HCDCE electrolyte) for aqueous zinc-ion batteries. Figure 7e illustrates the mechanism of HCDCE: the complexation between NH_4_Ac/NH_3_ and Zn^2+^ weakens Zn^2+^ solvation, reducing structural damage from electrostatic interactions during Zn^2+^ insertion/extraction. Additionally, the NH_4_^+^−NH_3_ buffer pair maintains stable electrolyte pH (Figure 7f,g), minimizing by-product formation and preserving electrochemical activity. This endows the material with exceptional cycling stability (4500 cycles with near-100% capacity retention, Figure 7h) and rate capability (Figure 7i).

Modification of electrolytes is achieved through three effective approaches: applying Le Chatelier’s principle by adding homologous salt ions to suppress Mn dissolution, utilizing high-concentration salt solutions to reduce Zn^2+^ solvation, and employing ionic buffer pairs to maintain pH stability. This strategy simultaneously addresses the challenges of eliminating Jahn–Teller distortion, suppressing manganese dissolution, and enhancing proton transfer kinetics, providing a new paradigm in aqueous zinc battery design.

## 4. Summary and Perspectives

In this review, we systematically studied the fundamental mechanisms and external triggers of Jahn–Teller distortions, analyzed the unfavorable effects on MnO_2_ cathodes, and provided targeted mitigation strategies. In AZIBs, while the MnO_2_-based host has achieved remarkable progress, the critical challenges for commercial implementation still need to be resolved. Then, we would suggest future prioritized research as the following directions:

First, it should deeply investigate the correlation between Jahn–Teller effects and charge storage mechanisms. Currently, the understanding remains incomplete due to the complex, condition-dependent reaction pathways observed in MnO_2_ cathodes. Therefore, the advanced in situ characterization platforms combining synchrotron X-ray diffraction, electrochemical mass spectrometry, and atomic force microscopy could provide unprecedented insights into real-time structural evolution and ion transport dynamics.

Second, we should focus on the design of comprehensive regulation strategies. Although Jahn–Teller distortion can arise from multiple factors, its fundamental origin lies in the intrinsic electronic structure of MnO_2_. Thus, cationic/anionic doping strategies that modulate electronic configurations play pivotal roles in suppressing Jahn–Teller distortion. Notably, the electrostatic interactions between Zn^2+^ and the host framework, coupled with structural degradation induced by water molecule intrusion, exacerbate Jahn–Teller effects. Consequently, integrating ion doping with interfacial engineering (e.g., pillar interlayer design) and electrolyte optimization to achieve multi-pronged suppression of Jahn–Teller distortion is critical for advancing MnO_2_-based electrodes. Firstly, synergistic doping-pillaring simultaneously tunes electronic structures and mitigates Zn^2+^-framework electrostatic forces. Secondly, doping-electrolyte coupling modifies charge/discharge mechanisms while optimizing electron delocalization.

Thirdly, research on by-products, intermediate products, and electrolyte additives should be strengthened. During the charge/discharge process, MnO_2_ cathode surfaces tend to generate by-products or intermediate products, which can affect battery performance and even alter the charge/discharge mechanisms. Currently, there is a lack of systematic studies on the types, structures, and influencing factors of these intermediate products. Electrolyte additives can effectively suppress surface-side reactions and stabilize material structures during charge/discharge cycles. While the effect of a single additive is limited, effective synergistic combinations of multiple additives may yield better results.

Fourth, Jahn–Teller distortion plays a critical role in battery systems utilizing MnO_2_-type octahedral frameworks as electrode materials, owing to structural deformation in transition metal complexes with octahedral coordination under specific electronic states to achieve energy minimization. This phenomenon manifests broadly across various battery systems, necessitating strategies to enhance the structural stability of manganese-based materials. Representative examples include suppression of Jahn–Teller distortion in LiMn_2_O_4_ spinel cathodes for Li-ion batteries, structural stabilization of layered NaMnO_2_ oxides for Na-ion batteries, and optimization of MnO_2_-based cathodes for Ca-ion batteries. Thus, mitigating Jahn–Teller distortion not only enhances the performance of zinc-ion battery cathodes but also serves as a universal strategy for advancing multivalent ion battery technologies.

## Figures and Tables

**Table 1:** Recent capacity retention and Mn dissolution of the cathode.

Cathode	Specific Capacity	Cycling Performance	Dissolved Mn^2+^	Refs.
δ-MnO_2_	125 mAh g^−1^ at 0.2 A g^−1^	14.3% after 200 cycles at 1 A g^−1^	2.5 mg L^−1^ after 50 cycles	[61]
α-MnO_2_/MGS	382.2 mAh g^−1^ at 0.3 A g^−1^	94% after 3000 cycles at 3 A g^−1^	0.42 mg L^−1^ after 1 cycle	[62]
Se-MnO_2_	386 mAh g^−1^ at 0.1 A g^−1^	78% after 5000 cycles at 3 A g^−1^	0.71 mg L^−1^ after 300 cycles	[63]
Al-MnO_2_	379 mAh g^−1^ at 0.2 A g^−1^	87% after 1000 cycles at 1 A g^−1^	0.12 mg L^−1^ after 50 cycles	[61]
δa-MnO_2_	175 mAh g^−1^ at 0.5 A g^−1^	91% after 500 cycles at 1 A g^−1^	0.54 mg L^−1^ after 100 cycles	[60]
Mn_2_O_3_@PPy	353.9 mAh g^−1^ at 0.5 A g^−1^	82% after 500 cycles at 1 A g^−1^	0.27 mg L^−1^ after 200 cycles	[64]
BMO	348 mAh g^−1^ at 0.1 A g^−1^	60% after 2000 cycles at 1 A g^−1^	0.015 mg L^−1^ after 100 cycles	[65]
δ-MnO_2_ (ZS-DOP electrolyte)	160 mAh g^−1^ at 1 A g^−1^	80% after 70 cycles at 7.5 mA g^−1^	1.2 mg L^−1^ after 100 cycles	[66]
AMO	400 mAh g^−1^ at 0.1 A g^−1^	94.5% after 2000 cycles at 2 A g^−1^	0.25 mg L^−1^ after 300 cycles	[67]

## Data Availability

No new data were created or analyzed in this study. Data sharing is not applicable to this article.

## Abbreviations

The following abbreviations are used in this manuscript:

SEM	Scanning electron microscope
TEM	Transmission electron microscope
HRTEM	High-resolution transmission electron microscope
SAED	Selected area electron diffraction
XRD	Phase analysis of X-ray diffraction
PVA	Polyvinyl alcohol
